# Supplementary material for: Inbreeding and Gallbladder Cancer Risk: Homozygosity Associations Adjusted for Indigenous American Ancestry, BMI, and Genetic Risk of Gallstone Disease
Source: Cancers (Basel). 2024 Dec 17;16(24):4195. doi: 10.3390/cancers16244195 (PMC11674764; doi:10.3390/cancers16244195)
Supplement: Supplementary file 1 [file cancers-16-04195-s001.zip › cancers-3272866-supplementary.pdf]

Supplementary table 1. Cohort characteristics according to ancestry, specifically the three components of north (Aimara-Quechua), south (Mapuche-Hulliche), and European ancestry. The classification thresholds were defined as follows: Aimara-Quechua (more than 70% of north ancestry component), A-Q-Europe (between 35% and 70% north ancestry component), European (more than 70% European ancestry), Mapuche-Huilliche (more than 70% south ancestry component), M-H-Europe (between 35% and 70% south ancestry component), and Other admixture (consisting of the remaining individuals).

	**All**	**Aymara-Quechua**	**A-Q-EU**	**Mapuche-Huilliche**	**M-H-EU**	**European**	**Other admixture**
N	4029	111	197	82	1885	113	1641
**Age**	37 (26-58)	25 (22-33)	25 (22-32)	43 (27-57)	44 (29-61)	39 (29-65)	34 (25-56)
**Sex (female)**	2284 (56.7%)	72 (64.9%)	115 (58.4%)	52 (63.4%)	1055 (56.0%)	70 (61.9%)	920 (56.1%)
**Education**							
Primary & informal schooling	515 (13.3%)	4 (3.6%)	0	34 (41.5%)	320 (16.9%)	4 (3.5%)	738 (44.9%)
Secondary	1749 (43.4%)	72 (64.9%)	134 (68.0%)	30 (36.6%)	742 (39.4%)	33 (29.2%)	153 (9.3%)
Postgraduate	69 (1.7%)	2 (1.8%)	4 (2.0%)	0	11 (0.6%)	12 (10.6%)	40 (2.4%)
Technical education	156 (3.9%)	5 (4.5%)	3 (1.5%)	1 (1.2%)	81 (4.3%)	6 (5.3%)	60 (3.6%)
University	532 (13.2%)	25 (22.5%)	55 (27.9%)	3 (3.7%)	119 (6.3%)	33 (29.2%)	297 (18.1%)
Missing	1008 (25.1%)	3 (2.7%)	1 (0.5%)	14 (17.1%)	612 (32.5%)	25 (22.1%)	353 (21.5%)
**BMI**	26.3 (23.7-29.6)	24.9(22.1-27.9)	25.5(23.1-29.4)	28.8(26.3-33.6)	26.8(25.1-30.2)	24.3(22.3-28.0)	25.9(23.5-29.2)
Genetic gallstone risk	0.445 (0.38-0.53)	0.449 (0.44-0.49)	0.449 (0.4-0.53)	0.449 (0.43-0.51)	0.445 (0.38-0.53)	0.43 (0.33-0.49)	0.445 (0.38-0.53)
**F_{ROH}**	0.009 (0.006-0.013)	0.028 (0.023-0.033)	0.011(0.007-0.014)	0.026(0.022-0.039)	0.01(0.006-0.013)	0.005(0.003-0.007)	0.007(0.005-0.011)

Supplementary table 2. OR and their 95% IC for Univariable and multivariable analysis of gallbladder cancer, stones and overweight. For multivariable analysis p-values for each variable is provided. Wald test was used to obtain the p-value of the overall effect of the education variable. OR FROH is given for an increase of 1%.

		Univariable logistic regression						Multivariable logistic regression								
		GBC		Gallstone disease		Overweight		GBC			Gallstone disease			Overweight		
**Variable**	**Level**	**OR**	**95% CI**	**OR**	**95% CI**	**OR**	**95% CI**	**OR**	**95% CI**	**P-value**	**OR**	**95% CI**	**P-value**	**OR**	**95% CI**	**P-value**
Aymara-Quechua	per 1%	**0.94**	0.92-0.95	**0.95**	0.93-0.96	**1.78**	1.95-0.10	**0.09**	0.09-0.10	0.03	**0.97**	0.96-0.98	6.7E-05	**1.00**	1.00-1.01	0.02
Mapuche-Huilliche	per 1%	**1.04**	1.03-1.05	**1.03**	1.02-1.04	**1.01**	1.00-1.02	**1.02**	1.01-1.03	1.6E-05	**1.01**	1.00-1.02	1.9E-04	**1.01**	1.00-1.01	0.0013
Gender	Male	Ref.		Ref.		Ref.		Ref.		2.1E-12	Ref.		2.1E-16	Ref.		0.05
	Female	**3.16**	2.55-3.94	**4.75**	3.92-5.78	0.95	0.82-1.10	**3.20**	2.40-4.20		**4.96**	3.95-6.26		0.83	0.70-0.98	
Age	per year	**1.06**	1.06-1.08	**1.05**	1.05-1.06	**1.02**	1.01-1.02	**1.31**	1.24-1.37	1.3E-16	**1.18**	1.14-1.23	2.1E-16	**1.12**	1.09-1.20	2.1E-16
Age^2	per $year^2$	**1.00**	1.00-1.00	**1.00**	1.00-1.00	**1.00**	1.00-1.00	0.99	0.97-1.00	2.6E-16	**0.99**	0.99-0.99	3.9E-14	**0.99**	0.99-0.99	7.3E-13
Education	Primary&Informal	**14.9**	11.4-19.7	**12.3**	9.40-16.2	**1.61**	1.25-2.09	**2.63**	1.87-3.71	3.5E-08	**3.16**	2.27-4.42	2.3E-04	0.97	0.70-1.33	0.07
	Secondary	Ref.		Ref.		Ref.		Ref.			Ref.			Ref.		
	Technical	**1.80**	1.03-3.04	**3.58**	2.31-5.49	**1.57**	1.00-2.53	**7.16**	3.84-1.28		**1.67**	1.01-2.73		1.15	0.72-1.87	
	University	**0.63**	0.42-0.92	0.99	0.73-1.33	0.89	0.72-1.09	**6.95**	4.46-1.06		0.95	0.68-1.31		0.86	0.69-1.07	
	Postgrade	0.43	0.11-1.23	0.81	0.33-1.69	1.20	0.70-2.09	**2.52**	5.84-7.48		**0.44**	0.17-0.98		0.95	0.55-1.68	
	Missing	0.77	0.56-1.06	**2.74**	2.20-3.41	**1.85**	1.52-2.28	**1.74**	1.21-2.49		0.87	0.67-1.14		**1.35**	1.07-1.70	
Genetic gallstone risk	Change per doubling of genetic risk	2.3	1.45-3.64	**2.97**	1.99-4.44	**1.21**	0.84-1.76	**2.61**	1.59-4.73	0.001	3.77	2.31-6.13	9.5E-08	**1.14**	0.78-1.73	0.487
FROH	per 1%	**3.56**	3.01-3.83	**3.25**	3.05-3.48	1.07	0.98-17.8	1.04	0.96-1.13	0.27	0.94	0.83-1.07	0.15	0.96	0.91-1.02	0.18

Supplementary table 3. OR, 95% IC and p values for gallbladder cancer presence split by sex. Wald test was used to obtain the p-value of the overall effect of education and BMI. OR FROH is given for an increase of 1%.

		Males			Females		
**Variable**	**Level**	**OR**	**95 CI**	**P-val**	**OR**	**95 CI**	**P-val**
**Age**	Per year	**1.45**	1.31-1.62	1.4E-14			
**Age2**	Per year2	**0.99**	0.96-0.99	2.6E-16			
**Education**	Primary & informal schooling	**1.81**	0.97-3.42		2.64	1.73-4.15	
	Secondary	Baseline		8.1E-05	Baseline		1.6E-16
	Technical education	**3.84**	1.07-12.2		0.31	0.13-0.67	
	University	**1.84**	0.89-3.69		0.40	0.22-0.69	
	Postgrad	**0.01**	0.00-11.4		0.16	0.02-0.59	
	Missing	**0.31**	0.15-0.63		0.12	0.07-0.18	
**BMI**	Normal	Baseline		0.2687	Baseline		2.6E-12
	Overweight	**0.81**	0.47-1.39		1.66	1.13-2.43	
	Obese	**1.28**	0.71-2.30		1.56	1.04-2.34	
**Ancestry**	Aimara-Quechua	**0.99**	0.96-1.02	0.6749	0.98	0.96-0.99	0.0513
	Mapuche- Huilliche	**1.02**	1.00-1.04	0.0372	1.03	1.01-1.05	3.1E-05
**Genetic gallstone risk**	Change per doubling of genetic risk	**3.51**	1.18-10.33	0.022	2.34	1.09-5.0	0.0271
**FROH**	Change per 1%	**1.19**	1.01-1.39	0.0021	1.05	0.94-1.18	0.3835

Supplementary table 4. OR, 95% IC and p values for gallbladder cancer presence split by age. Wald test was used to obtain the p-value of the overall effect of education and BMI. OR FROH is given for an increase of 1%.

		Age <= 60 years old			Age > 60 years old		
**Variable**	**Level**	**OR**	**95 CI**	**P-val**	**OR**	**95 CI**	**P-val**
**Sex**							
	Male	Baseline			Baseline		
	Female	**4.88**	3.27-7.43	2.9E-14	**2.15**	1.47-3.29	3.35E-04
**Age**	Per year	**1.15**	1.03-1.29	0.0111	**1.54**	0.77-3.17	0.2243
**Age2**	Per year2	**0.99**	0.97-1.00	0.3487	**0.99**	0.91-1.00	0.1694
**Education**	Primary & informal schooling	**8.75**	4.98-15.2		**0.95**	0.57-1.58	
	Secondary	Baseline		1.5E-12	Baseline		0.0784
	Technical education	**0.63**	0.28-1.03		**0.44**	0.08-2.17	
	University	**0.611**	0.34-1.05		**1.17**	0.46-3.07	
	Postgrad	**0.13**	0.007-0.21		**0.25**	0.01-2.17	
	Missing	**0.12**	0.005-0.20		**0.14**	0.07-0.25	
**BMI**	Normal	Baseline		0.0004	Baseline		0.2578
	Overweight	**1.37**	0.89-2.13		**1.00**	0.62-1.62	
	Obese	**2.29**	1.47-3.59		**0.75**	0.44-1.27	
**Ancestry**	Aimara-Quechua	**0.96**	0.94-0.98	0.0029	**1.03**	1.00-1.07	0.0364
	Mapuche- Huilliche	**1.02**	1.00-1.03	0.0058	**1.03**	1.01-1.05	8.3E-05
**Genetic gallstone risk**	Change per doubling of genetic risk	**3.47**	1.51-7.91	0.0031	**1.25**	0.46-3.39	0.6529
**FROH**	Change per 1%	**1.3**	1.09-1.98	0.0012	**0.89**	0.77-1.01	0.1259

Supplementary table 5. OR, 95% IC and p values for gallbladder stones presence. Wald test was used to obtain the p-value of the overall effect of education and BMI. OR FROH is given for an increase of 1%.

Variable	Level	OR	95 CI	P-val
**Sex**				
	Male	Baseline		
	Female	**4.99**	3.97-6.23	2.1E-16
**Age**	Per year	**1.20**	1.15-1.24	3.3E-14
**Age2**	Per year2	**1.00**	1.00-1.00	4.8E-13
**Education**	Primary & informal schooling	**3.11**	2.22-4.38	
	Secondary	Baseline		
	Technical education	**1.12**	0.64-1.93	
	University	**0.96**	0.68-1.35	
	Postgrad	**0.57**	0.12-1.12	
	Missing	**0.86**	0.38-1.24	
**BMI**	Normal	Baseline		4.4E-06
	Overweight	**1.93**	1.48-2.51	
	Obese	**1.25**	0.97-1.60	
**Ancestry**	Aimara-Quechua	**0.98**	0.97-0.99	1.6E-04
	Mapuche- Huilliche	**1.02**	1.01-1.03	6.9E-04
**Genetic gallstone risk**	Change per doubling of genetic risk	**4.01**	2.41-6.68	7.6E-08
**FROH**	Change per 1%	**0.96**	0.89-1.03	0.313
